# SNP Genotyping Characterizes the Genome Composition of the New Baisary Fat-Tailed Sheep Breed

**DOI:** 10.3390/ani12111468

**Published:** 2022-06-06

**Authors:** Narzhan Zhumadillayev, Kairat Dossybayev, Aigerim Khamzina, Tilek Kapasuly, Zhangylsyn Khamzina, Nurlan Tlevlesov

**Affiliations:** 1 Test Center, Kazakh Scientific Research Institute of Animal Husbandry and Forage Production, Zhandosov, 51, Almaty 050035, Kazakhstan; narzhan15@mail.ru (N.Z.); zh_khamzina@mail.ru (Z.K.); adairy@mail.ru (N.T.); 2 Laboratory of Genetics and Cytogenetics, RSE “Institute of Genetics and Physiology” CS MES RK, Al-Farabi Avenue, 93, Almaty 050060, Kazakhstan; tilek.kapas@mail.ru; 3 Department of Molecular Biology and Genetics, Faculty of Biology and Biotechnology, Al-Farabi Kazakh National University, Almaty 050040, Kazakhstan; 4 Green Biotechnology and Cell Engineering Laboratory, Kazakh National Agrarian Research University, Almaty 050010, Kazakhstan; aigerimkhamzina@mail.ru

**Keywords:** sheep, Baisary, SNP, Ovine SNP50K, genome and runs of homozygosity (ROH)

## Abstract

**Simple Summary:**

Historically, sheep breeding has played a key role in Kazakhstan. At present, due to the population’s increasing meat consumption, there is a high interest in the distribution of sheep breeds of different productivity levels. In our study, we describe the genetic structure and results of population analysis of a new breed called Baisary, which belongs to the meat-fat breeds and has favorable phenotypic traits. We reveal that this breed is genetically differentiated from its ancestors and other breeds, and shortly, it could be registered as a separate breed. This study helps with understanding the gene pool and genetic diversity of sheep breeds in Kazakhstan and should increase farmers’ interest in Baisary sheep.

**Abstract:**

Lamb meat has become increasingly popular in several nations during the last few decades, especially in Kazakhstan. Due to the rising demand for lamb meat, our sheep breeders developed a new fat-tailed sheep and named the breed Baisary. Animals of the Baisary breed are characterized by a large physique, strong constitution, stretched body, deep and wide chest, medium or large-sized fat tail, long legs (height at the withers of adult rams 85–100 cm, sheep 75–90 cm), long lanceolate ears and strong hooves. Lambs of the Baisary breed surpass their peers of the original parent breeds by 15–20% in live weight at the weaning period. To characterize the genetic structure of Baisary sheep and compare it with the ancestral breeds, we genotyped 247 individuals from five sheep breeds with Ovine SNP50K. The estimated private allelic richness ranged from 0.0030 to 0.0047, with the minimum and maximum provided by the Gissar (Giss1) and Kazakh meat-wool breeds, respectively. The highest and lowest F_IS_ values, meanwhile, were observed in the Afghan fat-tailed population and Baisary sheep, respectively. The calculated inbreeding coefficient showed that Edilbay and Baisary sheep have excess heterozygosity. According to principal components analysis, Baisary are close to Gissar populations, the Afghan fat-tailed breed and Edilbay sheep. These results were consistent with the Admixture and phylogenetic analysis. Overall, our results indicated that Baisary sheep differ genetically from their progenitors.

## 1. Background

Sheep breeding is one of the traditional occupations of the Kazakh people. Since ancient times, the life of Kazakhs has been directly related to the development of sheep breeding. It should be noted that Kazakhs prefer to breed fat-tailed sheep, for not only high-calorie dietary meat with a pleasant taste but also tail fat for use in the treatment of certain diseases [1]. Accordingly, meat-fat sheep breeds are in great demand in the country. Thanks to continuing efforts, several unique sheep breeds have been developed [2]. One of them is a new Ordabasy breed of meat-fat sheep (patent: Selection Achievement N282, 2013) [3]. This was created by a complex reproductive crossing of ewes of the local Kazakh coarse-wool fat-tailed breed with rams of Edilbay and Gissar sheep breeds, followed by breeding of the desired offspring (inter-se). The Ordabasy breed has meat and fat productivity. It is characterized by the following valuable productive and biological characteristics: early maturity and large live weight, strong constitution, coarse wool, taut fat-tail (large, medium, small), fitness and adaptability to desert, semi-desert and foothill breeding zones [3]. Currently, Kazakhstan farms more than 20 indigenous sheep breeds [4]. Each of the native sheep breeds has been studied for its phenotypic traits, and lately, studies have been published on the genetic characterization of the Kazakh indigenous sheep breeds using different molecular markers [4,5,6,7]. Four indigenous sheep populations (Edilbay, Kazakh fine wool, Kazakh Arkhar-Merino and Kazakh fat-tailed coarse wool) were examined using 12 STR loci, which were recommended by ISAG in 2014 and indicated all of the applied markers were high polymorphic. High genetic diversity was observed in all studied sheep populations except for Kazakh Arkhar-Merino [4,6]. Pozharskiy et al., who investigated Kazakh sheep breeds when compared with worldwide sheep based on the Ovine SNP50 BeadChip, concluded Edilbay sheep are direct descendants of historical domestic sheep ancestors [5]. When using seven STR loci, the analysis of genetic polymorphisms revealed low variability in Ordabasy and Karakul sheep breeds, which maintains their genetic diversity [7]. Molecular markers provide a greater understanding of the genetic structure and population background. SNP markers are the most powerful tools to assess genetic diversity and Admixture, either within or between breeds [8]. For instance, based on a genome-wide survey of SNP variation, genomic differences were established between the British Suffolk and two American Suffolk subpopulations, and the Australian Poll Dorset was genetically differentiated from American Dorsets [9,10]. The most widely used SNP panel for conducting genome-wide selection analysis, identifying quantitative trait loci (QTL), evaluating genetic variability and conducting linkage disequilibrium studies, comparative genetic studies and breed characterization, to evaluate the biodiversity among many sheep breeds, is the Ovine SNP50 BeadChip [11,12,13,14]. The BeadChip was developed by Illumina in collaboration with the International Sheep Genomics Consortium (ISGC) [11].

To date, the Baisary sheep breed is raised on several farms. Currently, there are over 9000 heads. The largest populations are in the Turkestan area on the Kuanysh and Nurbolat farms, the Zhambyl area on the Torekhan and Asan-Kerim farms and the Almaty area on the Kydyraliev and “Nur and K” farms.

When studying the growth and development of lambs at the ages of 4–4.5 months and 1.5 years, linear measurements of individual body parts were made to assess the external features of the Baisary breed. Between the two instances of measurement, the greatest intensity of growth was noted in the height at the withers and the oblique length of the body, along with the width, depth and girth of the chest.

In the present study, we present how this new sheep breed was developed, and we examine the genomic background of the Baisary breed using the Illumina Ovine SNP50 BeadChip. The article highlights how Baisary sheep are genetically differentiated from their ancestral sheep breeds.

## 2. Materials and Methods

### 2.1. Farm Locations

In late 1990, sheep breeders started a breeding program to create fat-tailed meat-lard sheep with high meat productivity on farms located in the Almaty, Zhambyl and Turkestan regions and the experimental farm within the Research Institute of Sheep Breeding, Medeubekov K.U, which is a branch of the LLP «Kazakh Research Institute of Animal Husbandry and Forage Production».

### 2.2. Mating Scheme

**Figure 1:**
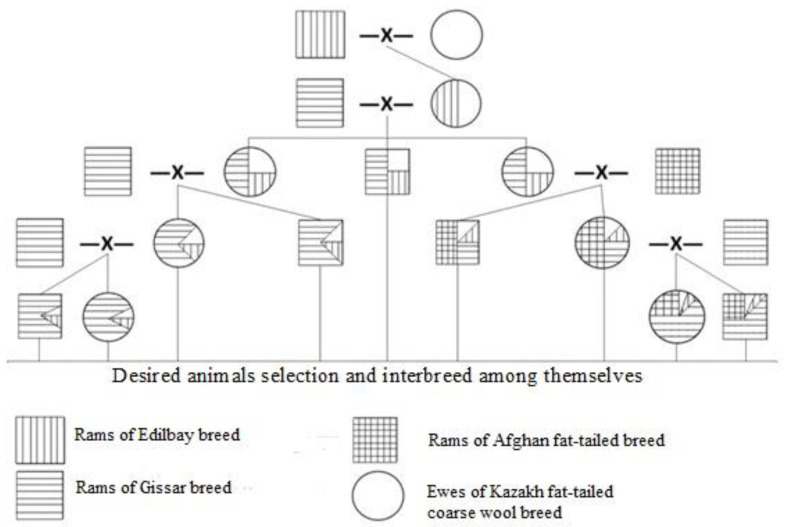
Breeding scheme for the Baisary breed of meat-fat sheep with improved productivity.

To develop the new breed (Figure 1), three breeds of ram participated in a mating scheme: Gissar, Afghan fat-tailed and Edilbay. All are bred for meat-fat production. Kazakh fat-tailed coarse-wool (KFTCW) ewes were crossed with the superior productive Edilbay (ED) rams. Then, individuals with a live weight of 60 kg or higher were selected from the available ewes. The first generation of KFTCW×ED crossbred animals mated with rams of Gissar and Afghan fat-tailed breeds. The progeny was interbred based on the selection of desired animals to produce the new breed (see Section 2.3). The breed was named Baisary. While breeding, reproductive crossing was carried out with appropriate choice of matched ewes and sires from the selected animals.

### 2.3. Animal Selection Criteria

Kazakh fat-tailed coarse-wool ewes were 2.5 years of age or older. The selected rams (Edilbay, Gissar and Afghan fat-tailed breeds) were at least 3.5 years old. Individuals with a live weight of 60 kg or more were selected from the dams. The live weights of the selected sires averaged (*n* = 2) 115 kg (Edilbay), 130 kg (Gissar) and 126 kg (Afghan fat-tailed). Sheep of the new breed are characterized by a high live weight, early maturity, excellent meat quality and adaptability to year-round transhumant pasture keeping. The animal selection criteria are given in Table 1.

### 2.4. Animal Sampling and SNP Genotyping

To study phenotypic traits, the live weight and six morphometric traits were measured for each animal. The morphometric trait measurements were: live weight, wither height, chest depth, chest width, body length, chest girth and loin girth. The morphometric traits were recorded on 6 Edilbay, 4 Gissar, 3 Afghan fat-tailed and 10 Baisary rams older than 2.5 years old, as well as 12 and 35 Baisary rams 1.5 and 4 months old, respectively.

A total of 247 samples were randomly selected for genotyping from five sheep populations: Edilbay (*n* = 55), Baisary (*n* = 96), Afghan fat-tailed (*n* = 38), Gissar (*n* = 38) and Kazakh meat-wool (*n* = 20). To extract whole-genome DNA, the ear tissue was collected from all selected animals. Genomic DNA was isolated from ear tissue specimens using MasterPure Complete DNA & RNA Purification Kits according to the manufacturer’s protocol and genotyped with Illumina Ovine SNP50 BeadChip microarrays using an iScan machine. PLINK text-format files were generated by PLINK Input Report Plug-in v2.1.4 for the Genome Studio Genotyping module. Additionally, Kyrgyz indigenous Aykol (*n* = 32) and Gissar (*n* = 30) sheep bred in Kyrgyzstan genotyped with Ovine SNP50 BeadChip datasets (available at Dryad https://doi:10.5061/dryad.37pvmcvf, (accessed on 31 May 2022)) were merged [15]. It was interesting to compare the Baisary breed with the Aykol sheep breeds because the Gissar breed participated in producing both. In this study, two populations of the Gissar breed were included: Gissar1 is from Tajikistan and Gissar2 is from Kyrgyzstan.

### 2.5. SNP Quality Control

Data quality control was performed using PLINK v1.90 software [16]. Markers unmapped to any chromosome, SNPs on the X and Y chromosomes and Mt SNPs were excluded from the dataset. SNPs with an allele call lower than 0.99 were omitted. The genotypes with a minor allele frequency (MAF) of less than 5%, markers that failed the Hardy–Weinberg test (*p* = 0.001), families with more than a 5% Mendel error rate and SNPs with more than a 10% Mendel error rate were omitted. Finally, a total of 44,140 out of 554,241 SNPs were retained after quality control, then due to missing genotype data, nine individuals of the Baisary breed were removed. Similar quality control procedures were performed for the merged dataset, and as a result of the filtering, 35,807 variants remained for our study. For population structure analysis, filtered data were pruned using PLINK based on the linkage disequilibrium (LD). LD pruning parameters were set as follows: (i) consider a window of 50 SNPs, (ii) remove one of a pair of SNPs if the LD is greater than 0.5 and (iii) shift the window five SNPs forward and repeat the procedure. After LD pruning, 25,275 markers were retained. Runs of homozygosity (ROH) were detected using PLINK v1.90 software [16], where the parameters and thresholds were set as follows: one SNP per 100 kb, 30 as the minimum number of SNPs in an ROH, 500 kb as the maximum gap between consecutive homozygous SNPs and 1 Mb as the minimum ROH length.

### 2.6. Genetic Diversity and Population Structure Analysis

To assess the genetic diversity within the population, the observed heterozygosity (Ho), unbiased expected heterozygosity (H_E(u)_) and inbreeding coefficient (F_IS_) were calculated by the R package “diveRsity” [17]. ADZE software [18] was applied to estimate the allelic richness (Ar) and private allele richness (pAr). Pairwise genetic differentiation F_ST_ values were estimated among the sheep populations using the ARLEQUIN program [19]. The phylogenetic tree was constructed from the matrix of pairwise F_ST_ values by the neighbor-joining method, as implemented in the program MEGAX [20]. Genetic structure analyses were performed in Admixture software [21], assuming *K* values ranging from 1 to 7. The optimal clusters were determined via calculation of the cross-validation (*CV*). The population structure was also examined through principal components analysis (PCA) in PLINK, using the option–pca, and visualized with GENESIS software [22]. PCA for morphometric traits was performed using the Past 4.03 program [23].

## 3. Results

### 3.1. Productive Qualities of a New Baisary Sheep of Meat-Fat Direction

The live weight of adult rams is 143 kg or more, ewes are 90–130 kg, four-month-old rams are 40–60 kg and ewe lambs are 45–56 kg. When weaning lambs of the Baisary breed, in terms of live weight, they surpass their peers of the original parent breed by 15–20%. Animals of the new breed are characterized by a large body type, strong constitution, elongated torso, deep and wide chest with a medium/large fat-tail size, long legs (height at the withers of adult rams 85–100 cm, ewes 75–90 cm), long lanceolate ears and strong hooves. Linear body measurements and live weights for the Baisary breed are given in Table 2.

**Figure 2:**
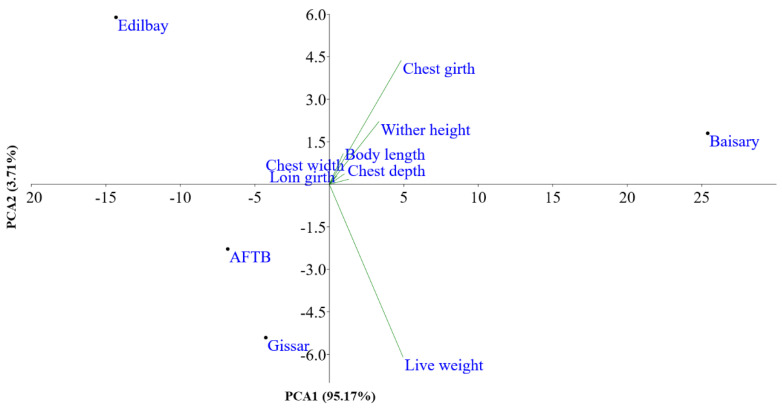
Principal components analysis of the physical traits of the sheep population.

PCA based on biometric traits was performed, and PCA1 and PCA2 were explained by 95.17% and 3.71% of the total variation, respectively (Figure 2). As such, PCA showed that the sheep breeds were clearly separated from each other. The first two PCA further demonstrated that all body measurements were clustered into one group except for the live weight. PCA1 showed high positive loadings for the live weight, wither height, chest depth, chest width and body length. PCA2 had high positive loadings, meanwhile, for the wither height, body length and chest girth.

### 3.2. Genetic Diversity Indices and Baisary Sheep Breed’s Relationships with Ancestral Breeds

The genetic diversity indices were estimated among sheep populations and breeds. The resulting summary statistics are given in Table 3. The allelic richness was similar for all populations except for Baisary sheep. Estimates of the private allelic richness were comparable among the sheep breeds, ranging from 0.0030 (Gissar1) to 0.0047 (Kazakh meat-wool breed). Interestingly, the Kazakh meat-wool breed had the highest private allelic richness values among all populations. To assess the genetic variation within breeds, we calculated the observed and expected heterozygosity. According to the mean observed heterozygosity, there was no difference between populations except for the Edilbay breed, while the expected heterozygosity varied from 0.38 in Baisary sheep to 0.4 in Afghan fat-tailed and Kazakh meat-wool sheep. The levels of inbreeding were examined by calculating the F_IS_ value for all populations. The Afghan fat-tailed population had the highest F_IS_ value, whereas the lowest F_IS_ value was found in the Baisary sheep. The inbreeding coefficient results revealed that excess heterozygosity was observed in the Edilbay and Baisary sheep.

A total of 32,878 ROH segments were observed in the five sheep populations. Descriptive statistics for the ROH are presented in Table 4. The highest average number of ROH was noted in Gissar1 (145.13 ± 8.28), whereas the lowest was observed in KMWB (120.6 ± 18.7). The mean ROH lengths varied from 213.6 ± 13.94 Mb in the ED breed to 267.23 ± 60.77 Mb in the Baisary breed. The maximum ROH number per animal was found in Gissar1 and the minimum was recorded in the KMWB breed. In all studied populations, the longest individual ROH length was identified in the Gissar1 breed (602.31 Mb) and the shortest was observed in ED (574 Mb).

**Figure 3:**
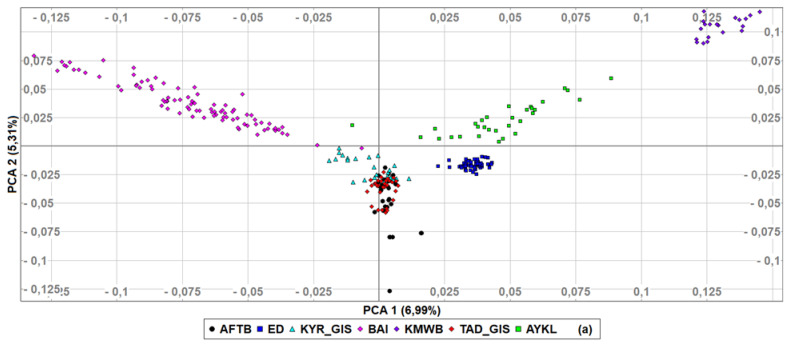

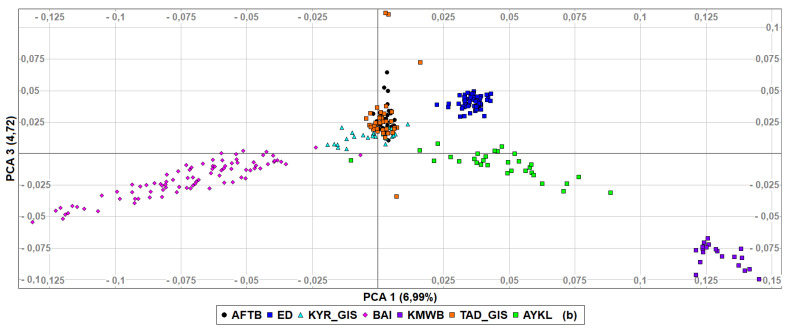
Principal components analysis of the studied sheep populations (**a**) PCA1 and PCA2, (**b**) PCA1 and PCA3. Afghan fat-tailed breed (AFTB), Aykol (AYKL), Edilbay (ED), KYR_GIS (Gissar breed—imported from Kyrgyzstan), Baisary (BAI), Kazakh meat-wool breed (KMWB), TAD_GIS (Gissar breed—imported from Tajikistan).

To determine in detail the genetic relationships among populations based on genotyped data, first, we performed PCA (Figure 3) with Kazakh meat-wool sheep added as an outgroup. The first principal component explained 6.99% of genetic variance, with two Gissar populations and AFTB very closely related, while Kazakh meat-wool sheep were genetically distinct, set apart from other populations. PCA2 then accounted for 5.31% of the diversity, with Baisary sheep clearly differentiated as a breed set apart from all other breeds/populations. According to the third component, which explained 4.72% of the genetic diversity, the Baisary sheep were closer to the Gissar and Afghan fat-tailed populations than the Aykol and Kazakh meat-wool breeds. The first two PCAs revealed that all breeds formed non-overlapping clusters, except for Gissar and Afghan fat-tailed sheep. The individuals within each breed clustered closely together, and Edilbay sheep were observed as the most consolidated breed.

**Figure 4:**
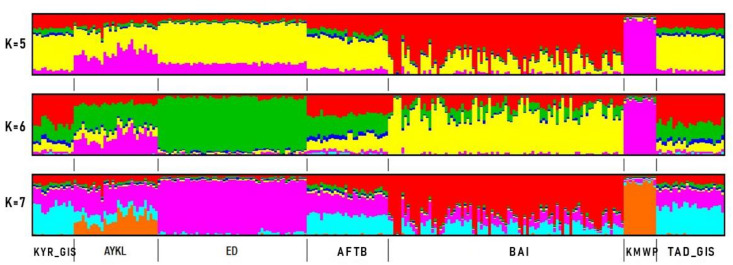
Assessment of population genetic structure in different sheep breeds. Afghan fat-tailed breed (AFTB), Aykol (AYKL), Edilbay (ED), KYR_GIS (Gissar breed—imported from Kyrgyzstan), Baisary (BAI), Kazakh meat-wool breed (KMWB), TAD_GIS (Gissar breed—imported from Tajikistan).

Furthermore, to examine the extent of Admixture (Figure 4) between breeds/populations, standard Admixture analyses were conducted. The optimal number of assumed ancestral populations was observed at *K* = 5, 6 and 7, respectively (Figure S1). The results of cluster analysis demonstrated that at *K* = 5, Kazakh meat-wool breed sheep were clearly differentiated from all others. The Kazakh meat-wool sheep contribution was found in the Aykol breed. At *K* = 5, 6 and 7, Edilbay sheep had the uniform genetic profile of their own genome, and the Edilbay genome pattern was found in all populations. According to *K* from 5 to 7, the two Gissar sheep populations and Afghan fat-tailed sheep were assigned to the same clusters. Despite being a new sheep breed, the Baisary breed was significantly differentiated from those of its ancestral origins. Patterns from Gissar populations predominated in the Baisary breed compared to Edilbay at 6 and 7 *K* values.

We also estimated the pairwise F_ST_ values to assess the genetic relatedness among populations. In the resulting pairwise F_ST_, Kazakh meat-wool sheep were the most divergent breed. The two Gissar and Afghan fat-tailed populations were not genetically differentiated from each other based on the genotyped data. When considering pairwise F_ST_ calculations, the largest genetic distance was found between the Kazakh meat-wool and Baisary breeds, and the second-highest F_ST_ value observed between Edilbay and Kazakh meat-wool sheep. The calculated F_ST_ value indicated that the Baisary sheep are closer to the Gissar2 population than the Aykol and Edilbay breeds. The F_ST_ value was lowest between the Aykol and Kazakh meat-wool breeds compared to Afghan fat-tailed, Gissar2, Baisary and ED populations (Table S1).

**Figure 5:**
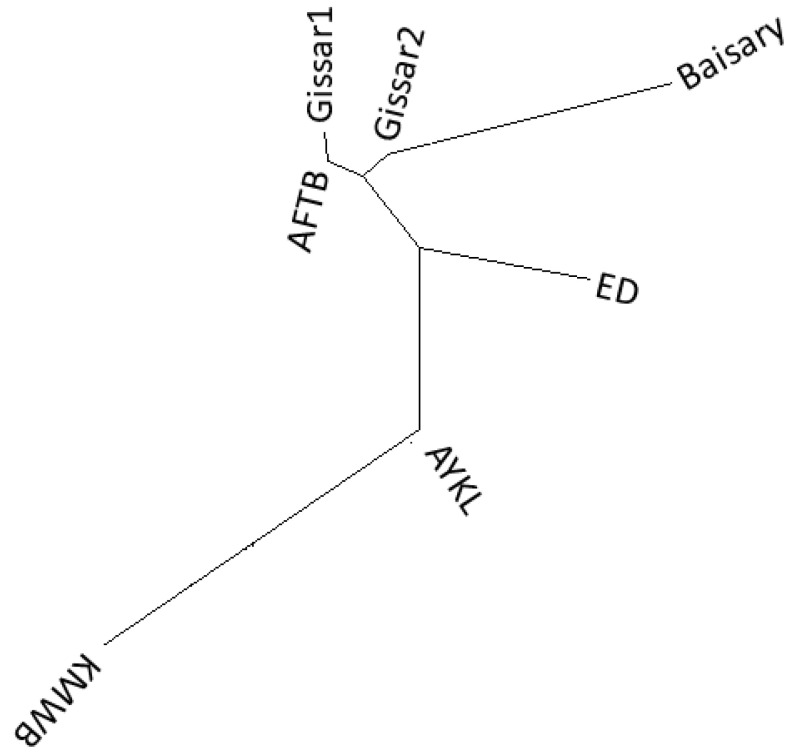
Phylogenetic relationship of sheep breeds based on genetic F_ST_ distances. Afghan fat-tailed breed (AFTB), Aykol (AYKL), Edilbay (ED), Gissar2 (imported from Kyrgyzstan), Baisary (BAI), Kazakh meat-wool breed (KMWB), Gissar1 (imported from Tajikistan).

A phylogenetic tree (Figure 5) was constructed based on the pairwise F_ST_ genetic distances by using the neighbor-joining method to represent the relationships of studied populations. The sheep populations were divided into two groups: one for the Afghan fat-tailed and Gissar sheep populations, and the other for the Aykol and Kazakh meat-wool breeds. The Edilbay sheep breed was positioned between these two groups. Afghan fat-tailed, Gissar1 and Gissar2 populations were sub-clustered together and grouped with Baisary sheep on the same node.

## 4. Discussion

Due to consumers’ ever-increasing demand for lamb meat, we must develop new breeds of sheep characterized by high meat productivity and early maturity during the milk-feeding period of development and up to 16–18 months of age. These requirements are fully satisfied by fat-tailed lambs and young sheep of the meat-fat direction. Based on these considerations, the goal was set to create a new breed of fat-tailed meat-fat sheep with high meat productivity (Figure S2. A typical ram and a typical ewe). The live weight of the resulting Baisary breed of lambs is 15–20% greater at weaning when compared to the original parental breeds. Similar results were reported by Azhimetov et al., who found that the live weights of the crossbred sheep were higher than those of the parental breeds [24]. Our results are in agreement with several studies, for example, Zonabend investigated the phenotypic traits of lambs of the Red Maasai and Dorper breeds and their crosses, finding that the live weights of crosses were heavier compared to both parental pure breeds [25]. Similar results were also reported by Gebreyowhens et al., Momani et al. and Dawson et al. [26,27,28,29].

Assessments of genetic diversity are a key aspect in the development of sustainable breed-improvement strategies, conservation programs and adaptations to extreme environmental conditions [30]. To determine the genetic diversity of the studied breeds, we estimated the distributions of alleles across populations, along with the observed and expected heterozygosity. The allelic richness was similar in all studied populations and comparable to the results reported by Deniskova et al. [15]. As such, our results infer that the newly produced sheep population’s long-term potential for adaptability and persistence is not differentiated from that of ancestor breeds. The Baisary sheep breed was formed from different fat-tailed breeds, which have the main advantage of being highly adapted to the environment, for instance, the Afghan fat-tailed meat-fat sheep breed is distinguished by its endurance and adaptation to a variety of climatic conditions [31]. The Gissar breed of sheep, meanwhile, is adapted to flat and mountainous conditions and has good endurance, while Gissar rams can easily adapt to new conditions. Gissar sheep were raised by nomadic people in harsh environmental conditions with year-round stays on pastures, meaning they have good feeding qualities [32]. Edilbay and Kazakh fat-tailed coarse-wool breeds, meanwhile, are adapted to harsh winter frosts and summer droughts. Edilblay sheep are the most ancient breed in Kazakhstan and show good adaptability to living in all zones of the country [4]. 

The private alleles are useful to examine the population structure and migration across populations [33,34]. The estimated number of private alleles was similar in all populations except for the Kazakh meat-wool breed. All breeds that participated in producing the new breed are in the meat-fat and coarse-wool directions. Compared to other breeds, the highest mean number of private alleles was observed in the Kazakh meat-wool breed. According to Kalinowski (2004), when several populations in a sample are closely related and populations come from a common origin, few alleles are private to individual populations [35]. The results of our tests for genetic variability showed that random mating occurs in Edilbay and Baisary sheep populations. The inbreeding coefficient of individuals in subpopulations also demonstrated excess heterozygotes in both populations. These results are in agreement with a previous study where Kazakh sheep breeds were examined based on the 12 microsatellite loci, with the highest genetic diversity observed in Edilbay sheep [4]. Considering F_IS_ values, the largest inbreeding coefficient was for Afghan fat-tailed sheep and indicated a reduction in heterozygosity due to non-random mating. The longest mean ROH length (267.23 ± 60.77 Mb) was observed in Baisary sheep. The Baisary breed was formed under stronger selective pressure when selecting individuals while fixing the breed. These results are consistent with findings for Romanov (282 Mb) sheep, which are also a coarse-wool breed. According to Deniskova, the Romanov breed was formed under great selective pressure from individuals with the best pelt qualities and prolificacy [36].

PCA clustered two Gissar sheep populations with Afghan fat-tailed sheep breed, which matches their geographic distribution. According to PCA, the Baisary sheep are close to Gissar and Afghan fat-tailed sheep, though the Baisary sheep remain distinct from all other breeds in PCA despite the newness of the breed. The official breed formation of Baisary sheep began in late 1990 and had long generation intervals, and the PCA results could be interpreted to confirm that Baisary sheep have become established as genetically different from their progenitors. The Admixture results confirmed that the genetic pattern of the Edilbay breed was found in all sheep. Edilbay is considered the most ancient breed in our country, and due to its superior meat production, is used to improve meat productivity in other sheep breeds raised in Kazakhstan and abroad [15]. Admixture analysis revealed that Baisary sheep share significant genetic background with Gissar sheep, more so than Edilbay, and these results are in agreement with the breeding scheme. The highest F_ST_ value was found between the new sheep and Kazakh meat-wool breed because this breed did not participate in developing the Baisary sheep. The Kazakh meat-wool breed was also the most divergent among all the studied populations. It is characterized by fine-wool sheep, whereas all the other examined breeds are coarse-wool sheep. The pairwise F_ST_ values were consistent with the phylogenetic relationship inference. Interestingly, the phylogenetic tree showed that Kazakh meat-wool breed sheep were clustered with the Aykol breed, which might be explained by the origin of Aykol sheep; this breed resulted from crossing local fat-rumped and local fine-wool sheep with Gissar rams [15]. The neighbor-joining tree indicated the Baisary sheep were grouped with Gissar and Afghan fat-tailed sheep breeds, which is consistent with the origin of the Baisary breed.

## 5. Conclusions

This is the first study to describe the genetic structure of the newly created Baisary sheep breed and its ancestral breeds using Ovine SNP50K markers, as well as phenotypic traits. Our results may be used as a foundation to investigate fat-tailed sheep breeds on the genomic level and develop sustainable programs to conserve the genetic diversity of Baisary sheep in the future. The results showed that Baisary sheep are genetically differentiated as a breed from their progenitors. PCA demonstrated that Baisary sheep score well in six body measurements (wither height, chest depth, chest width, body length, chest girth and loin girth). In the future, these traits could be used to rank animals when evaluating the meat-fat tailed sheep breeds.

## Figures and Tables

**Table 1:** Measurements of the rams used to create the breed.

Measurements	Edilbay (ED *n* = 6)		Gissar (Giss1 *n* = 4)		Afghan Fat-Tailed Breed (AFTB *n* = 3)	
	Mean ± SE	σ	Mean ± SE	σ	Mean ± SE	σ
Live weight	115.0 ± 1.24	3.03	130.0 ± 0.41	0.81	126.0 ± 0.58	2.64
Wither height	86.5 ± 0.85	2.07	87.7 ± 0.75	1.50	87.0 ± 0.57	1.00
Chest depth	39.0 ± 0.93	2.28	40.5 ± 0.29	0.57	39.7 ± 0.33	0.58
Chest width	24 ± 1.06	2.61	24.5 ± 0.75	0.58	25.3 ± 0.67	1.15
Body length	90 ± 0.73	1.79	89.8 ± 0.85	1.71	89.3 ± 1.20	2.08
Chest girth	107 ± 0.73	1.79	106.8. ± 0.48	0.96	107.3 ± 0.88	1.53
Loin girth	9.22 ± 0.30	0.75	9.33 ± 0.25	0.5	9.7 ± 0.88	1.53

**Table 2:** Measurements and live weights of the Baisary breed at the experimental farm in the Kazakh Institute of Sheep Breeding, K.U. Medeubekov.

Measurements	Adult Rams (*n* = 10)		1.5 Year-Old Rams (*n* = 12)		4 Month-Old Rams (*n* = 35)	
	Mean ± SE	σ	Mean ± SE	σ	Mean ± SE	σ
Live weight	143 ± 3.33	5.77	78 ± 2.91	5.88	41.4 ± 0.74	4.38
Wither height	102.0 ± 1.53	2.64	86.25 ± 1.32	2.63	71.9 ± 0.71	4.20
Chest depth	45.3 ± 0.67	1.15	35.5 ± 0.65	1.29	26.9 ± 0.45	2.69
Chest width	29.0 ± 0.58	1.0	22.8 ± 1.43	2.87	17.9 ± 0.27	1.57
Body length	94.0 ± 3.06	5.29	86.8 ± 3.90	7.80	59.4 ± 0.60	3.53
Chest girth	129 ± 2.64	4.58	102.5 ± 1.85	3.7	78.6 ± 0.62	3.68
Loin girth	11.6 ± 0.33	0.57	10.3 ± 0.25	0.5	9.5 ± 0.60	0.79

**Table 3:** Indices of genetic diversity of five sheep populations.

Breeds	*n*	Ar	pAr	Ho	He	F_IS_ (Cl = 95%)
Afghan fat-tailed breed (AFTB)	38	1.99	0.0034	0.39	0.4	0.0043 (−0.026 to 0.0073)
Edilbay (ED)	55	1.99	0.0036	0.4	0.39	−0.0115 (−0.0254 to −0.017)
Baisary (BAI)	87	1.98	0.0034	0.39	0.38	−0.0099 (−0.0233 to −0.0068)
Kazakh meat-wool breed (KMWB)	20	1.99	0.0047	0.39	0.4	0.0023 (−0.0517 to 0)
Gissar (Giss1)	38	1.99	0.0030	0.39	0.39	0.0026 (−0.0266 to −0.00080)

Number of individuals (*n*), allelic richness (Ar), private allele richness (pAr), observed heterozygosity (Ho), unbiased expected heterozygosity (He), inbreeding coefficient (F_IS_).

**Table 4:** Descriptive statistics for runs of homozygosity for five sheep breeds.

Breed	*n*	Total Number of ROH	ROH Length			ROH Number		
			Mean	Min.	Max.	Mean	Min.	Max.
AFTB	38	5288	233.171 ± 56.45	143.56	503.64	139.16 ± 14.2	91	165
ED	55	7493	213.6 ± 13.94	176.54	284.6	136.23 ± 6.05	121	153
BAI	87	12,170	267.23 ± 60.77	165.42	597.55	139.89 ± 7.2	118	160
KMWB	20	2412	229.84 ± 71.94	145.58	574	120.6 ± 18.7	96	152
Gissar1	38	5515	259.46 ± 73.54	183.5	602.31	145.13 ± 8.28	128	169

Afghan fat-tailed breed (AFTB), Edilbay (ED), Baisary (BAI), Kazakh meat-wool breed (KMWB), Gissar1 (imported from Tajikistan).

## Data Availability

Not applicable.

## Supplementary Materials

The following supporting information can be downloaded at: https://www.mdpi.com/article/10.3390/ani12111468/s1, Figure S1. A graph should be given: cross-validation error vs. K, Figure S2. A typical ram and a typical ewe, Table S1. Population pairwise differentation in between breeds.
