# Supplementary material for: SNP Genotyping Characterizes the Genome Composition of the New Baisary Fat-Tailed Sheep Breed
Source: Animals (Basel). 2022 Jun 6;12(11):1468. doi: 10.3390/ani12111468 (PMC9179407; doi:10.3390/ani12111468)

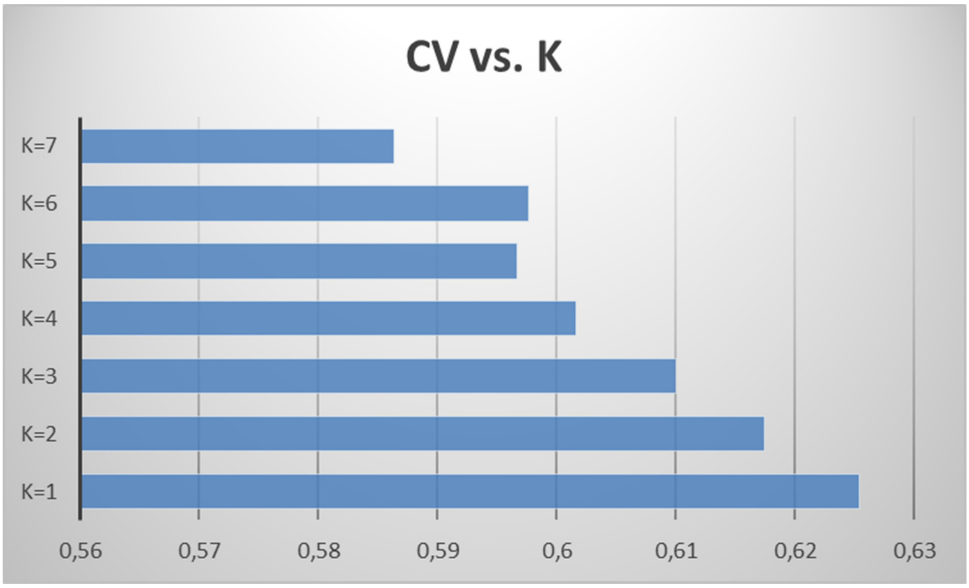

Figure S1.A graph should be given: cross-validation error vs. K.

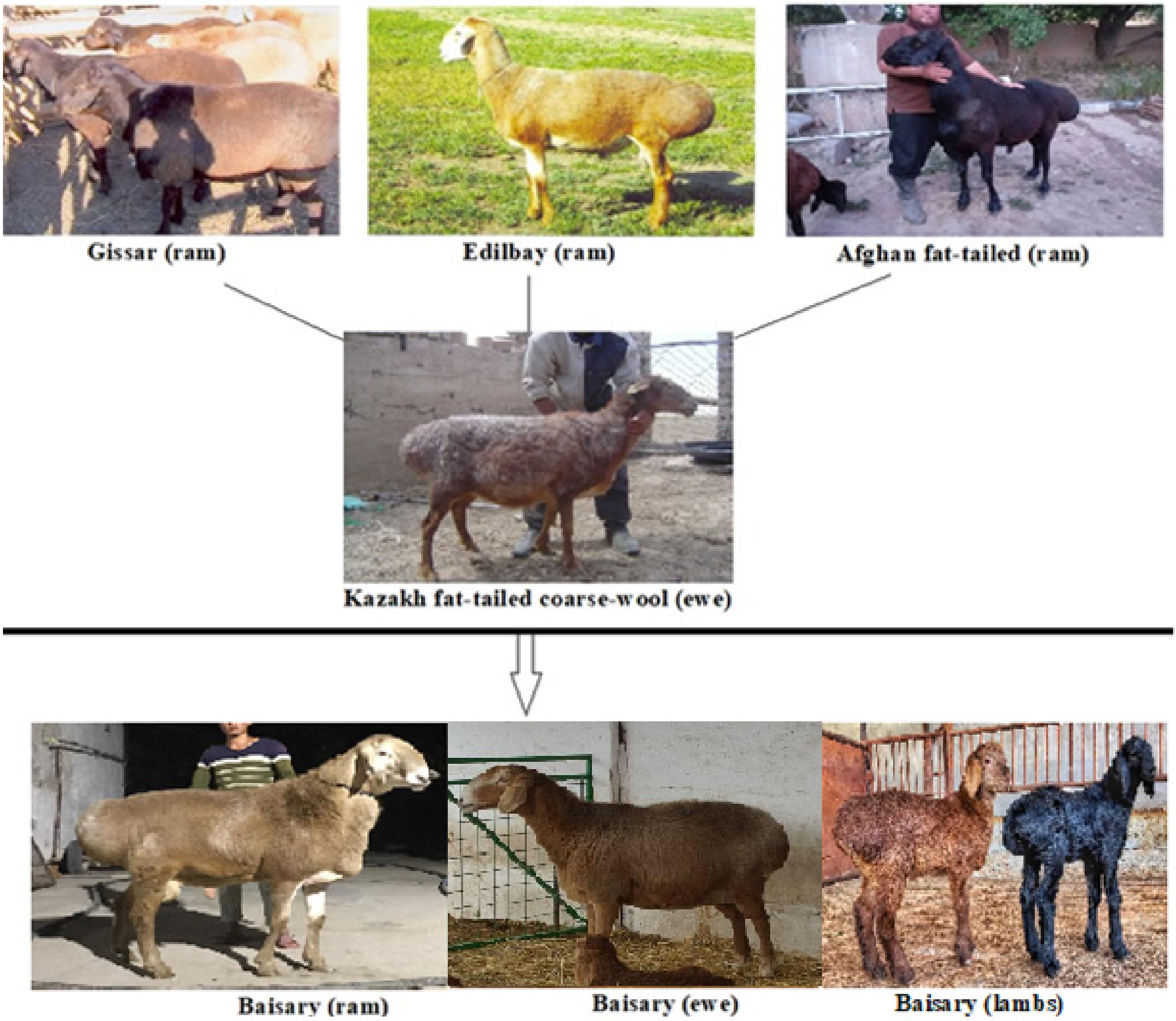

Figure S2. A typical ram and a typical ewe. Photo by Zhumadillaev N., Rustemov A. and Mykhtybayev K.

Table S1. Population pairwise differentation in between breeds

Pop ID	AFTB	AYKL	ED	Gissar2	Baisary	KMWB	Gissar1
AFTB	**0.00000**						
AYKL	0.00286	**0.00000**					
ED	0.00608	0.00462	**0.00000**				
Gissar2	-0.00182	0.00203	0.00499	**0.00000**			
Baisary	0.00767	0.00996	0.01397	0.00533	**0.00000**		
KMWB	0.01444	0.00551	0.01733	0.01493	0.02346	**0.00000**	
Gissar1	-0.00419	0.00520	0.00819	-0.00019	0.00968	0.01695	**0.00000**

Afghan fat-tailed breed (AFTB), Aykol (AYKL), Edilbay (ED), Gissar2 (imported from Kyrgyzstan), Baisary (BAI), Kazakh meat-wool breed (KMWB), Gissar1 (imported from Tajikistan)

Additional pictures of Baisary sheep breed

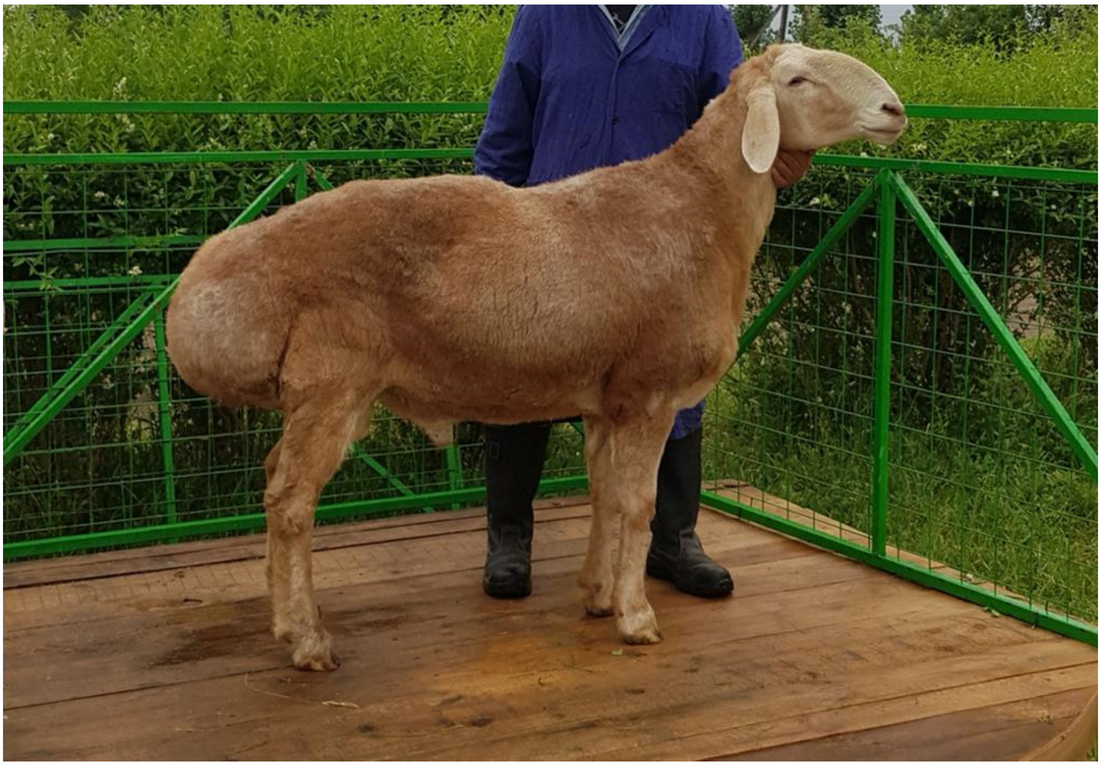

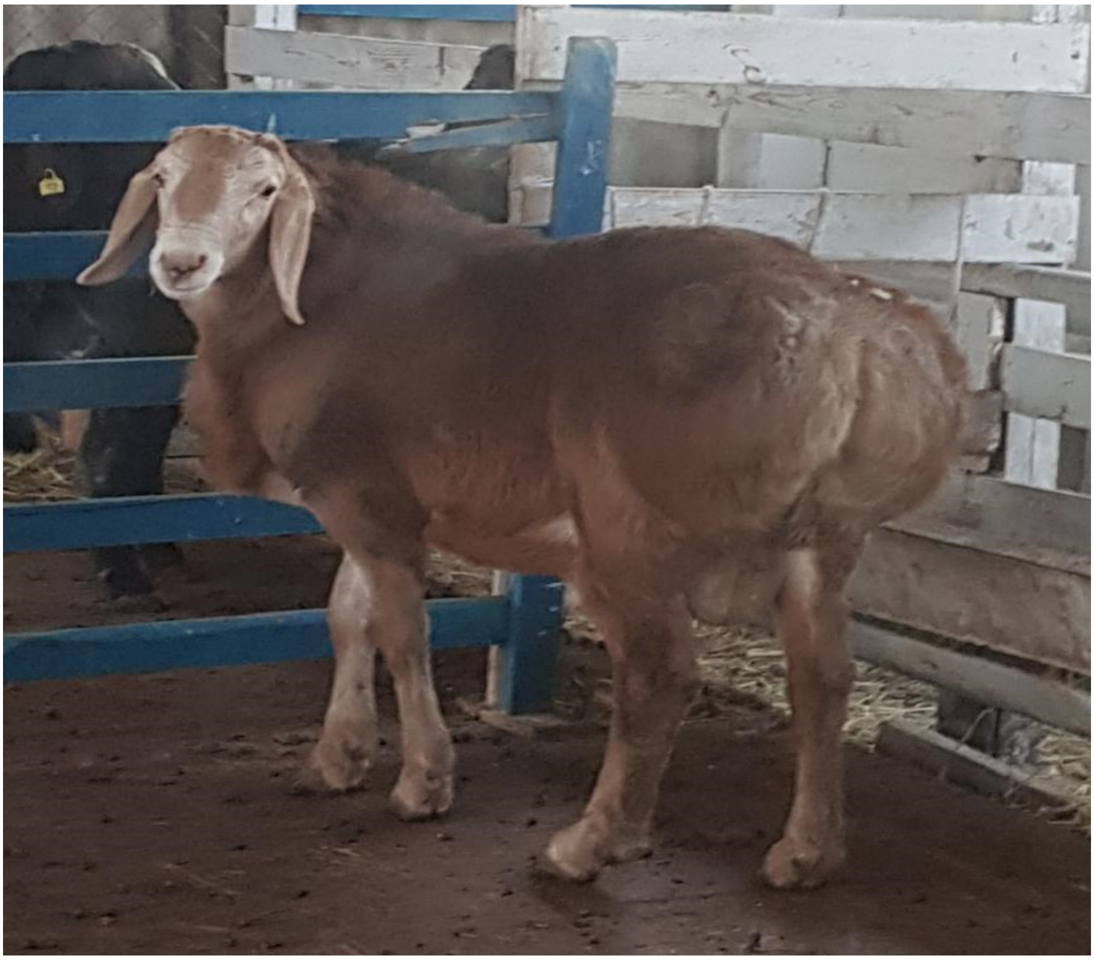

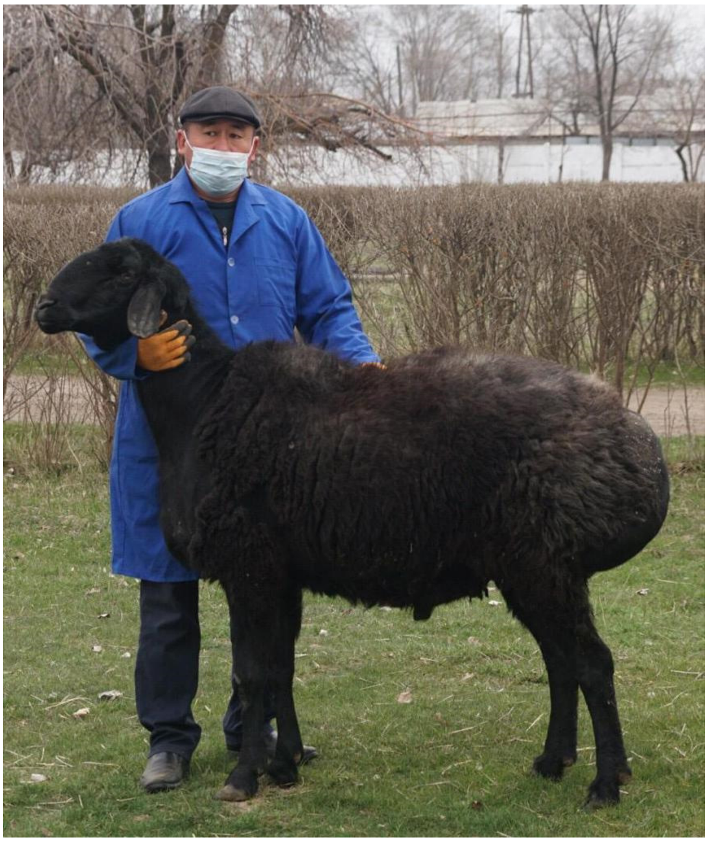

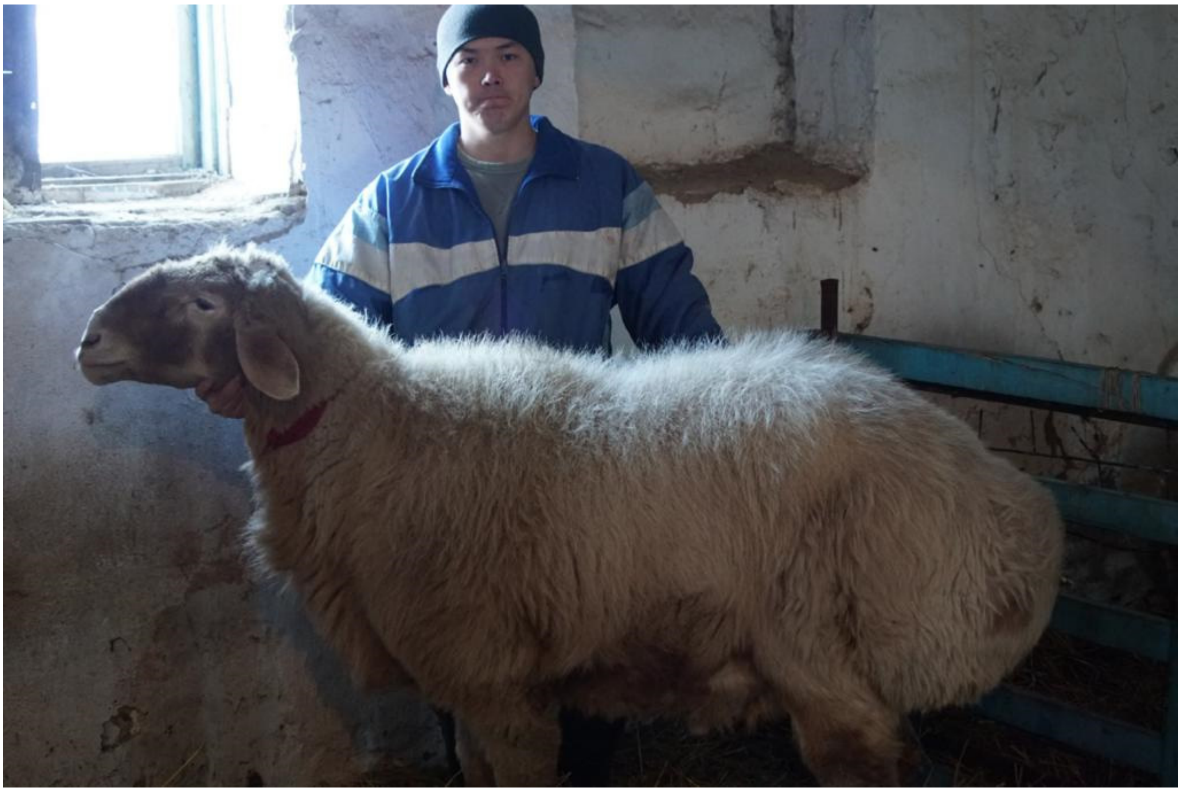

Supplement: Supplementary file 1 [file animals-12-01468-s001.zip › animals-1614384-supplementary.pdf]
